# Structure and function of a lignostilbene-α,β-dioxygenase orthologue from *Pseudomonas brassicacearum*

**DOI:** 10.1186/s12858-018-0098-4

**Published:** 2018-08-16

**Authors:** Peter C. Loewen, Jacek Switala, James P. Wells, Fang Huang, Anthony T. Zara, John S. Allingham, Michele C. Loewen

**Affiliations:** 1 Department of Microbiology, University of Manitoba, Winnipeg, MB R3T 2N2 Canada; 2 National Research Council of Canada, 100 Sussex Drive, Ottawa, ON K1A 0R6 Canada; 3 Department of BioMedical and Molecular Sciences, Queen’s University, Kingston, ON K7L 3N6 Canada

**Keywords:** Carotenoid, Crystal structure, In silico modeling, Lignostilbene cleaving oxygenase, Resveratrol

## Abstract

**Background:**

Stilbene cleaving oxygenases (SCOs), also known as lignostilbene-α,β-dioxygenases (LSDs) mediate the oxidative cleavage of the olefinic double bonds of lignin-derived intermediate phenolic stilbenes, yielding small modified benzaldehyde compounds. SCOs represent one branch of the larger carotenoid cleavage oxygenases family. Here, we describe the structural and functional characterization of an SCO-like enzyme from the soil-born, bio-control agent *Pseudomonas brassicacearum*.

**Methods:**

In vitro and in vivo assays relying on visual inspection, spectrophotometric quantification, as well as liquid-chormatographic and mass spectrometric characterization were applied for functional evaluation of the enzyme. X-ray crystallographic analyses and in silico modeling were applied for structural investigations.

**Results:**

In vitro assays demonstrated preferential cleavage of resveratrol, while in vivo analyses detected putative cleavage of the straight chain carotenoid, lycopene. A high-resolution structure containing the seven-bladed β-propeller fold and conserved 4-His-Fe unit at the catalytic site, was obtained. Comparative structural alignments, as well as in silico modelling and docking, highlight potential molecular factors contributing to both the primary in vitro activity against resveratrol, as well as the putative subsidiary activities against carotenoids in vivo, for future validation.

**Conclusions:**

The findings reported here provide validation of the SCO structure, and highlight enigmatic points with respect to the potential effect of the enzyme’s molecular environment on substrate specificities for future investigation.

## Background

Lignostilbene-α,β-dioxygenases (LSDs), also known as stilbene cleaving oxygenases (SCOs), catalyze the cleavage of the double bonds of intermediate stilbenes (e.g. resveratrol), yielding small modified benzaldehydes [1]. SCO enzymes are one branch of a much larger family that also includes carotenoid cleavage oxygenases or dioxygenases (CCOs or CCDs) as well as 9-*cis*-epoxycarotenoid dioxygenases (NCEDs). These are best known for their oxidative cleavage of double bonds of large straight chain, acyclic and bicyclic carotenoids, yielding an array of smaller molecules collectively called apocarotenoids [2]. These apocarotenoids play important biological roles in a wide array of organisms and form a diverse family of naturally occurring metabolites, including vitamin A [3], plant hormones such as abscisic acid [4, 5], saffron spice and the pigment bixin (annatto) [6], as well as smaller, volatile, β-ionones and damascones, which provide the aromas in tea, grapes, roses, tobacco and wine [7].

CCOs form part of a larger superfamily of non-heme, Fe-dependent oxygenase enzymes [8, 9]. CCOs generally have multiple carotenoid substrates, but possess high regio- and stereo-selectivity for cleavage sites along the substrate polyene chain. To date, the structures of four different CCOs, including an apocarotenoid oxygenase from *Synechocystis* sp. 6803 (ACO; PDB: 2BIW) [10], a retinoid isomerase, RPE65 from *Bos taurus* (PDB: 3FSN) [11], the NCED VP14 from *Zea mays* (PDB: 3NPE) [12] and, most recently, an SCO from *Novosphingobium aromaticivorans*, (NOV1; PDB: 5 J53) [13] have been reported. Despite varied amino acid sequences, the enzymes share a common seven-bladed β-propeller fold that orients the four fully conserved, Fe-coordinating His residues in the catalytic center, adjacent to which a hydrophobic substrate-binding site is formed by a helix-loop-containing domain [14].

*Pseudomonas brassicacearum* is a soil-born γ-proteobacteria that functions as a biocontrol agent against the plant pathogenic fungus *Sclerotinia sclerotiorum* [15, 16]. Its mode of bio-control has been linked to a variety of mechanisms involving the production of an array of secreted bio-control factors, including degradative enzymes, hydrogen cyanide (HCN), and a novel anti-fungal lipopeptide called sclerosin [16, 17]. Its genome encodes an orthologue of known bacterial LSDs, which is documented for the first time herein, and referred to as *P. brassicacearum* lignostilbene dioxygenase (*Pb*LSD). Generally, the expression of LSD’s in non-carotenogenic bacteria is associated with enabling their symbiotic interactions with host plants, contributing to the digestion of plant-derived lignocellulosic material into smaller metabolites to use as sources of nutrients. However many of the potentially derived mono-phenolic compounds, eg ferulic acid, have known anti-fungal activity and thus LSD’s may also contribute to the mechanisms underlying the biocontrol functionality of the bacteria.

In this report we further describe the characterization of *Pb*LSD as catalyzing the cleavage of the model stilbene, resveratrol, in vitro and with in vivo evidence of very weak putative cleavage activity against both straight chain and bi-cyclic carotenoids. The X-ray crystallographic structure determined for *Pb*LSD highlights the conservation of the expected seven-bladed β-propeller fold and 4-His-Fe unit, characteristic of CCOs. Comparative structure alignments and in silico docking of various substrates identify aspects of the *Pb*LSD substrate binding cavity that may be contributing to the enzyme’s substrate specificities as documented herein. Indeed the differences in in vitro versus in vivo assay outcomes emphasize a need to better understand the relationship between these enzymes and their environment, as it pertains to oligomerization, membrane localization, substrate accessibility and specificity.

## Methods

### Materials

All chemicals and reagents were purchased from Sigma-Aldrich unless otherwise indicated.

### Phylogenetic analysis

The amino acid sequences were obtained using the accession numbers (indicated in the figure legend) from the NCBI (National Center for Biotechnology Information) web site. Sequences were aligned using Clustal Omega [18] and their evolutionary relationship investigated using Mega6 [19].

### Cloning of *Pb*LSD and *At*CCD1 (*Arabidopsis thaliana* carotenoid cleavage dioxygenase 1)

An open reading frame (ORF) clone of *Pb*LSD, identical in sequence to that reported in the NCBI data base (Uniprot W8QAY8–1; NCBI CP007410; WP_025212951), was obtained by PCR amplification from the genomic DNA of *P. brassicassearum* [20]. Primers used included Forward-A 5’-GTGATGAGGGTACCATATGAGTATTCCTTT-3′ or Forward-B 5’-GTGAGCAACTAGTATGAGTATTCCTTTTCC-3′ and Reverse 5’-GGGAGGGATTGGATCCTGTCAGGAACCCGG-3′, for introduction of *Kpn*I and *Nde*I (Forward A) or *SpeI* (Forward B) restriction sites immediately ahead of the gene and a *Bam*HI site immediately following the stop codon. The amplified ORFs were subsequently cloned into the pET28b + expression vector at the *Nde*I and *Bam*HI sites, producing an expression construct (pET28b + -*Pb*LSD) encoding an N-terminally His-tagged *Pb*LSD fusion protein; and into the pET41a + expression vector at the *SpeI* and *BamHI* sites, producing a construct (pET41a + -*Pb*LSD) encoding an N-terminally GST-tagged *Pb*LSD fusion protein. *At*CCD1 (Uniprot O65572) was cloned as described previously [21, 22].

### Recombinant *Pb*LSD activity in carotenoid-accumulating strains of *E. coli*

For functional studies the spectroscopic method as described by Schwartz et al. [22], was used as follows. The pET28b + -*Pb*LSD construct was co-transformed into *E. coli* BL21 (DE3) cells with one each of the carotenoid accumulating plasmids pAC-BETA, pAC-DELTA, pAC-EPSILON, pAC-LYC, and pAC-ZEAX (Addgene plasmids # 53272, 53,273, 53,276, 53,270, 53,274 respectively) [23–26]. To achieve this, 2 mL cultures were grown overnight in 2YT medium (per liter: 16 g of tryptone, 10 g of yeast extract, and 5 g of NaCl) with 30 mg/mL kanamycin and 35 mg/mL chloramphenicol. The overnight cultures were used to inoculate (1:50 ratio) 30 mL cultures of 2YT with the same antibiotics and grown for 24 h at 18 °C in the dark. Protein production was induced with the addition of 0.1 mM isopropyl-ß-D-thiogalactopyranoside (IPTG) and ferrous sulfate to a final concentration of 10 mg/L and cultures further incubated for 48 h at room temperature in the dark. For quantitative analysis of carotenoid accumulation, 1 mL of each culture was centrifuged, and the medium was discarded. The cell pellets was each resuspended in 100 μL of formaldehyde, and then 1 mL of ethanol was added. Samples were incubated at 4 °C for 3 h before the cell debris was removed by centrifugation. The resulting supernatants were analyzed for carotenoid content. For ß-carotene- and zeaxanthin-accumulating strains of *E. coli*, absorbance was measured with a Lambda35 spectrophotometer (GE) at 450 nm. For δ- and ε- carotene strains, carotenoid was measured at 460 nm. For the lycopene-accumulating strains, absorbance was measured at 470 nm. The carotenoid content was calculated using extinction coefficients with units of (g/100 ml)^− 1^ cm^− 1^, as follows: 2620 for β-carotene, 2540 for zeaxanthin, 3120 for ε - carotene, 3290 for δ - carotene, 3450 for lycopene, and plotted relative to controls that included un-induced (no IPTG) co-transformed cells, and IPTG-induced cells transformed with only the carotenoid accumulating plasmid (no pET28b + -PbCCO).

### Expression and purification of recombinant *Pb*LSD and *At*CCD1

For the purposes of in vitro assays and protein crystallization, *E. coli* BL21 (DE3) was transformed with either pET41a + -*Pb*LSD or pET28b + -*Pb*LSD respectively. Transformed colonies were grown overnight at 37 °C in LB media (per liter: 8 g of tryptone, 5 g of yeast extract, and 5 g of NaCl) containing either 100 mg/mL ampicillin or 30 mg/mL kanamycin as needed.

For in vitro assays, the resulting pET41a + -*Pb*LSD transformed culture was used to inoculate one 5 mL culture (1: 100 ratio; 2YT media + 100 mg/mL ampicillin). The culture was grown at 37 °C to an optical density at 600 nm (OD_600_) of 0.6. Protein expression was then induced by the addition of 1 mM IPTG, and the cultures were incubated at 28 °C for an additional 16 h. The pellets were suspended in 50 mM sodium phosphate buffer pH 7.0 with 1 mM EDTA and 1% Triton X-100. Lysozyme was added to a final concentration of 1 mg/ml with 0.1 mM PMSF. The mixture was incubated for 30 min at 4 °C on an end-over-end shaker. The cells were sonicated for 20 s intervals on ice, at 30% maximal power until a clear lysate was achieved. The solution was clarified by centrifugation. The resulting supernatant was applied to Glutathione Hicap Matrix (Qiagen, Hildon, Germany) and the GST-*Pb*LSD fusion protein was purified following the manufacturer’s instructions using 50 mM NaH_2_PO_4_, 150 mM NaCl, pH 7.2, 1 mM DTT and 1 mM EDTA as the equilibration and wash buffer. Protein was eluted with 50 mM Tris pH 8.0, 0.4 M NaCl, 50 mM reduced L-Gluthathione, 0.1% Triton X-100 and 1 mM DTT. Eluted fractions containing *Pb*LSD were pooled, and the buffer was exchanged to 100 mM Tris-HCl, pH 7.0, 0.1% Triton X-100 while the protein was concentrated by microfiltration. *At*CCD1 was produced and purified as described previously [21, 22].

For crystallization, the resulting pET28b + -*Pb*LSD transformed culture was used to inoculate 2 × 500 mL cultures in fluted flasks (1:100 ratio; LB media + 30 mg/mL kanamycin). Cultures were grown at 37 °C to an OD_600_ of 0.6. Protein expression was then induced by the addition of 0.1 mM IPTG, and the cultures were incubated at 28 °C for an additional 16 h. The pellets were resuspended in 50 mM potassium phosphate (KPi) pH 7.0 with 1 mM EDTA. Lysozyme was added to a final concentration of 1.2 mM and the mixture incubated for 5 min at 37 °C with stirring and another 5 min at room temperature, and then cooled on ice. After 2–4 passages through a French Press cell at 20,000 psi, the solution was clarified by centrifugation. Streptomycin sulfate was added to the clarified supernatant to a final concentration of 2.5% to precipitate out any DNA, and the mixture stirred for 20 min at 4 °C after which the precipitate was removed by centrifugation. The protein solution was subjected to ammonium sulfate fractionation, with the bulk of the recombinant *Pb*LSD precipitating in the 40 and 45% ammonium sulfate fractions. These protein pellets were dissolved in 50 mM KPi pH 7.0 on ice and dialyzed overnight against 50 mM KPi pH 7.4 at 4 °C. The fusion protein was further purified by nickel-NTA affinity chromatography using a HiTrap Chelating HP column (GE Healthcare) following the manufacturer’s instructions using 20 mM KPi, 0.5 M NaCl, 5 mM imidazole pH 7.4 as the binding buffer and eluting with 20 mM KPi, 0.5 M NaCl, 500 mM imidazole pH 7.4. Eluted fractions containing *Pb*LSD were pooled, concentrated by microfiltration and dialyzed into 50 mM KPi pH 7.0 to remove any residual imidazole. The purified protein was then stored at − 60 °C.

### In vitro activity assay of *Pb*LSD and *At*CCD1

Assays were performed essentially as described previously [27]. Purified proteins (50–100 μg) were added to 400 μl reactions containing phosphate buffer (50 mM, pH 7.2), NaCl (300 mM), sodium ascorbate (10 mM) and FeSO_4_ (0.5 mM), or 100 mM Tris-HCl pH 7.0, 0.1% Triton X-100, sodium ascorbate (5 mM) and FeSO_4_ (0.5 mM) for resveratrol or lutein containing-reactions respectively. Resveratrol (1 mM final concentration; Sigma-Aldrich; from a 1 M stock dissolved in dimethyl sulfoxide (DMSO)) or lutein (0.1–0.2 μM; purified by HPLC from spinach and dissolved in 0.1% Triton X-100) were used in the reactions. In vitro reactions were carried out at 30 °C for 2 h in the darkness. Reactions were stopped by extraction with ethyl acetate (800 μL). The organic fractions were dried under a stream of nitrogen and stored at − 20 °C for analysis.

Thin layer chromatography: substrate and products in the reaction extractions were separated on a thin-layer silica gel 60 W plate (Sigma-Aldrich) and developed in hexane, ethyl acetate, and 2-propanol (70:20:10). Following chromatography, the plate was sprayed with 2,4-dinitrophenylhydrazine to detect aldehydes and ketones. The extractions were also analyzed on an Agilent 1100 series HPLC system with a Diode array detector and quaternary pump solvent system (Agilent, Palo Alto, CA). A 3.9 × 300 mm Waters μBondapak C18 column (Waters, Milford, MA) was used for analysis. To detect resveratrol-derived in vitro reaction products, a solvent system of water:trifluoroacetic acid (99.9:0.1, *v*/v) (A) and methanol:trifluoroacetic acid (99.9:0.1, v/v) (B), and a gradient program was used: from 0 to 10 min 75:25 A:B, followed by a gradient from 75:25 A:B to 50:50 A:B in 15 min, followed by 13 min at 50:50 A:B, with a flow rate of 0.5 mL/min and monitored at 452 and 290 nm. For analyzing lutein-derived in vitro reaction products, mobile phase solvents consisted of deionized water (A), 100% acetonitrile (B) and 100% acetone (C) and a gradient program with 50% acetonitrile and water at 1 mL/min for 8 min followed by a linear gradient to 100% acetonitrile over 42 min. The gradient was then shifted to 100% acetone over 20 min and left at 100% acetone for an additional 10 min. For mass spectroscopic analysis, samples were dissolved in 100% acetonitrile. The mass spectra were monitored in a mass range of *m/z* 100–500 using an electrospray ionization interface in the positive mode with SQ Detector 2 (Waters, Milford, MA). The flow injection was used and the solvent system consisted of deionized water and acetonitrile (50:50, *v*/v). The flow rate was 25 μL/minute. No separation was attempted.

### Crystallization and structural elucidation of *Pb*LSD

The purified enzyme was crystallized by vapor-diffusion in hanging-drops using the EasyXtal Tool (Qiagen) 15-well plates and supports. One μL of protein solution (10 mg/mL *Pb*LSD in 50 mM KPi pH 7.0) was mixed with an equal volume of reservoir solution containing 24–26.5% PEG 3350 and Bis-Tris 100 mM pH 6.5. The mixed solution drop was then equilibrated against 750 μL of reservoir solution at room temperature. Crystals were harvested into mother liquor with 20% glycerol as cryoprotectant and stored in liquid nitrogen for X-ray diffraction data collection using synchrotron beam line CMCF 08ID-1 at the Canadian Light Source in Saskatoon, SK. Data were processed and scaled using XDS [28] and SCALA [29] (Table 1). Starting with a single chain from the structure of *Synechocystis* apocarotenoid-15,15′-oxygenase (PDB: 2BIW), the data were phased with MOLREP [30] revealing four subunits in the asymmetric unit, and the refinement was completed using the program REFMAC [31] and manual modeling with the molecular graphics program COOT [32]. The unit-cell parameters and processing statistics are included in Table 1. Figures were generated using PYMOL [33]. The structure has been deposited with PDB ID: 5V2D.

**Table 1:** Data collection and refinement statistics of PbLSD - PDB Accession # 5V2D

A. Data collection statistics	
Space group	P2_1_
a (Å)	96.40
b (Å)	104.67
c (Å)	104.71
β (°)	94.79
Resolution^a^	48.03–1.90 (2.00–1.90)
Unique reflections	161,021 (23,680)
Completeness %	99.0 (99.8)
R_merge_	0.046 (0.495)
R_pim_	0.031 (0.341)
<I/σI>	16.1 (2.5)
CC(1/2)	0.999 (0.780)
Multiplicity	3.1 (3.0)
B. Model refinement statistics	
No. reflections	152,884
R_cryst_ (%)	16.1
R_free_ (%)	19.4
Non-H atoms	16,443
Water Molecules	1075
Average B-factor Å^2^	
Protein	34.7
Waters	36.5
Other	
Coor. err. Å^b^	0.098
rms dev. Bonds Å	0.023
rms dev. Angles ^o^	2.07

^a^Values in parentheses correspond to the highest resolution shell

^b^Based on maximum likelihood

### In silico modelling and docking

Glide 5.0 was used for soft receptor molecular docking through the Maestro software suite [34]. The receptor grids for *Pb*LSD were prepared using the OPLS_2005 force field. While active site water molecules were excluded from grid generation, the His-coordinated Fe(II) ion was included. As a means of softening the potential for non-polar parts of the receptors, atomic van der Waals radii were scaled by a factor of 0.8. Atoms were considered for scaling if their absolute partial atomic charge was determined to be ≤0.25, indicating them as non-polar. As a final step in grid generation, rotations were allowed for all applicable sidechains. The ligand array was prepared using LigPrep [35] and isomerization about polyene double bonds was restricted. The prepared ligands were docked into the generated receptor grids using Glide XP docking with flexible ligand sampling. For this procedure, a second softening potential was applied, except with regard to the ligands. A 0.8 scaling factor was used for van der Waals radii of atoms within each ligand that maintained absolute partial atomic charges ≤0.15. All poses were subjected to post-docking minimization. The best-docked structures were considered for each ligand based on the GlideScore, a metric calculated by Glide 5.0 as an estimation of binding free energy.

## Results and discussion

### Phylogenetic analysis of the LSD homologue from *P. brassicacearum* (*Pb*LSD)

**Fig. 1:**
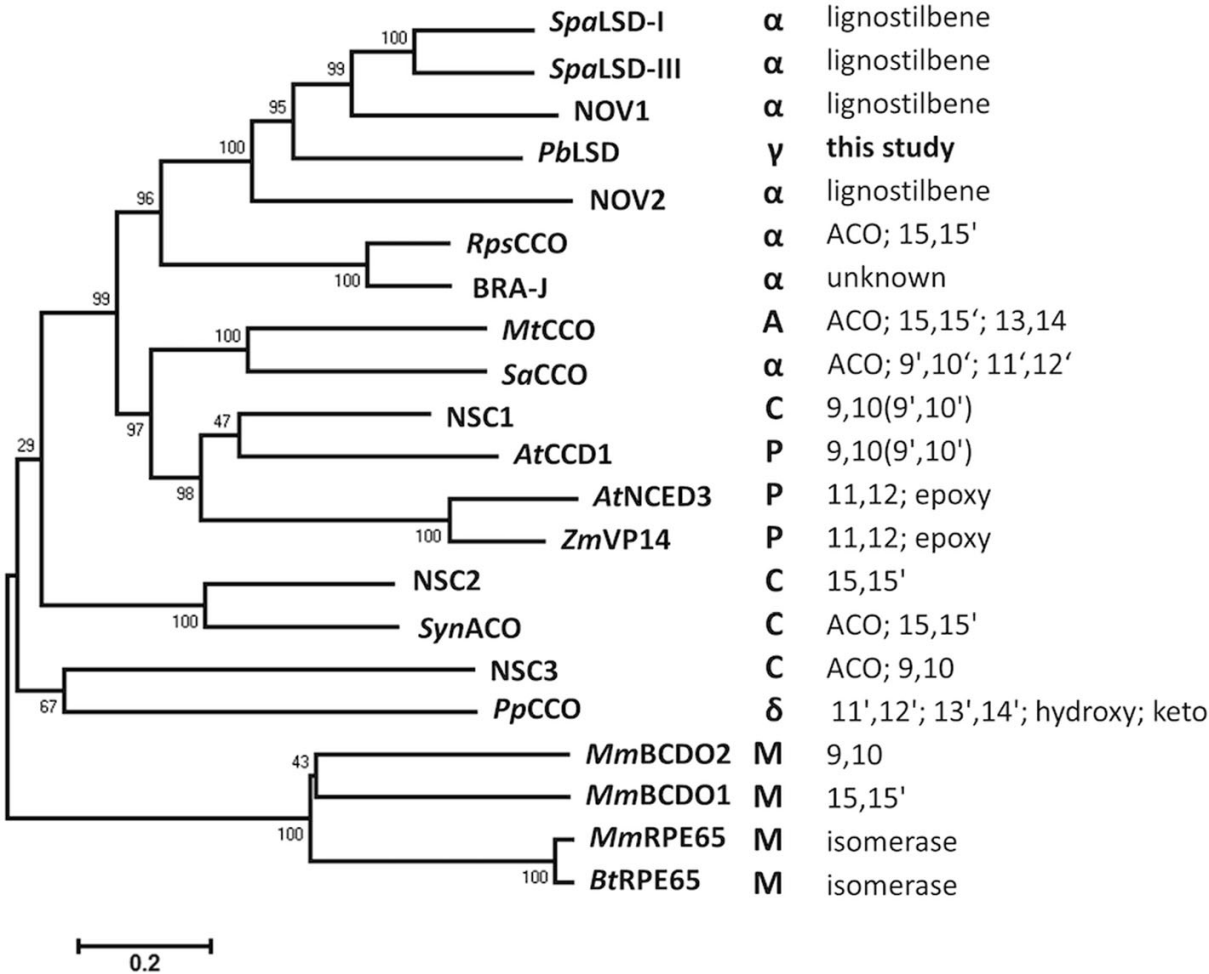
Evolutionary relationships and functional summary of select microbial homologs of carotenoid cleavage oxygenases, including plant and mammalian references. The evolutionary history was inferred using the Neighbor-Joining method [35]. The optimal tree with the sum of branch length = 8.88 is shown. The tree is drawn to scale, with branch lengths in the same units as those of the evolutionary distances used to infer the phylogenetic tree. The evolutionary distances were computed using the Poisson correction method [41] and are in the units of the number of amino acid substitutions per site. The analysis involved 21 amino acid sequences. All positions containing gaps and missing data were eliminated. There were a total of 346 residues in the final dataset. Evolutionary analyses were conducted in MEGA6 [42]. The notations are as follows: **α** - α-proteobacteria, **δ** - δ –proteobacteria, **γ** - γ-proteobacteria, **C** - cyanobacteria, **A** - Actinobacteria, **P** - plants, and **M** - mammal. The right most column highlights information about known substrates and cleavage sites. ACO refers to apocarontenoids substrates (mono-cyclic). If no substrate is indicated, C40 carotenoid substrates (either acyclic or bicyclic) can be assumed. Sequence sources: *P. brassicacearum Pb*LSD (WP_025212951), *S. paucimobilis Spa*LSD-I (AAC60447) & SpaLSD-III (AAB35856); *N. aromaticivorans* NOV1 (WP_011444461) and NOV2 (WP_011446449); *R. palustris Rps*CCO (WP_011156772); *B. japonicum* BRA-J (NP_772430); *M. tuberculosis Mt*CCO (P9WPR4), *S. alaskensis Sa*CCO (WP_011541991), *Nostoc sp.* PCC 7120 NSC1 (WP_010995279), NSC2 (WP_010998422) and NSC3 (WP_010999021); *Synechocystis sp.* PCC 6803 SynACO (WP_010873049); *A. thaliana At*CCD1 (NP_191911), *At*NCED3 (NP_188062), *Z. mais Zm*VP14 (NP_001105902), *P. pacifica Pp*CCO (ZP_01913312), *M. musculus Mm*BCDO2 (Q99NF1), *Mm*BCDO1 (Q9JJS6) and *Mm*RPE65 (Q91ZQ5), *B. taurus Bt*RPE65 (NP_776878)

*Pb*LSD was initially identified on the basis of its amino acid similarity to other CCOs including 48% amino acid identity to the *S. paucimobilis* LSD-I (AAC60447.2) and 35% identity to the *R. palustris* CCO (WP_011156772). To determine its fit in the larger oxygenase family, a phylogenetic analysis was performed including microbial, plant and mammalian CCOs, which placed *Pb*LSD within a group of microbial LSD proteins (Fig. 1).

### In vitro enzymatic activity of recombinant PbLSD

**Fig. 2:**
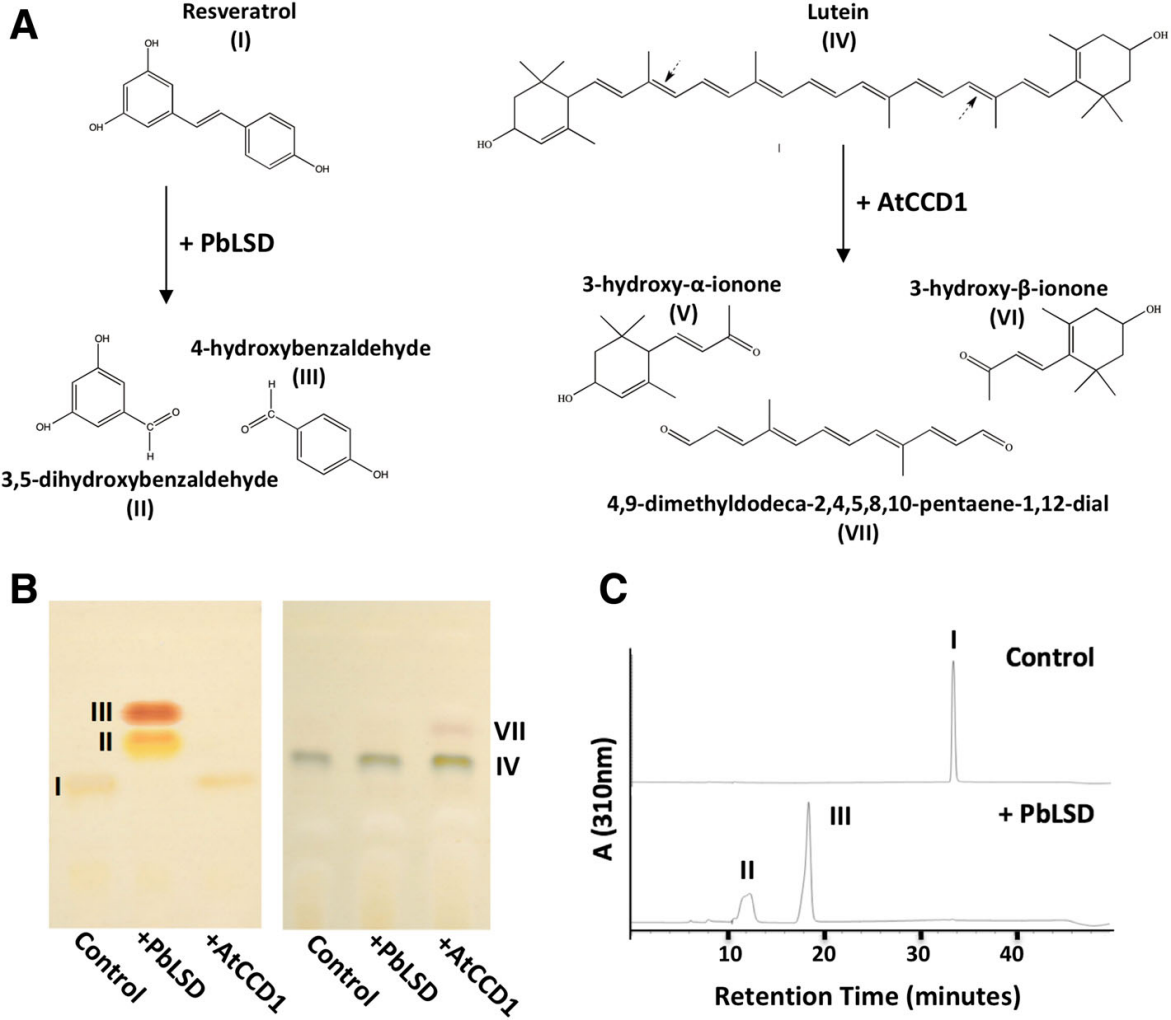
In vitro enzymatic activity of recombinant *Pb*LSD on resveratrol and lutein (**a**) Left panel: Structure of the stilbene resveratrol (I) and the products, 3,5-dihydroxybenzaldehyd (II) and 4-hydroxybenaldehyde (III) produced by the enzymatic activity of *Pb*LSD. Right panel: Structure of the carotenoid lutein (IV) and the products, 3-hydroxy-α-ionone (V), 3-hydroxy-β-ionone (VI) and 4,9-dimethyldodeca-2,4,5,8,10-pentaene-1,12-dial; C14 dialdehyde; (VII)) (**b**) Thin-layer chromatography analysis of assays with the recombinant *Pb*LSD and *At*CCD1 proteins when applied to either resveratrol (left panel) or lutein (right panel) substrates. Assignment of products arising from resveratrol was based on the expectation that the additional hydroxyl group on compound II yields a higher polarity compound, increasing its tendency to stay in the more hydrophobic solid phase, thus slowing its migrate compared to compound III. Migration of the expected C14 dialdehyde (compound VII) product arising from the reaction of *At*CCD1 on lutein is consistent with previous observations [22]. The products produced by recombinant *Pb*LSD from resveratrol were further characterized by reverse-phase HPLC. The expected products are labeled on the chromatogram again based on retention of the less polar compound III in the more hydrophobic stationary phase, with compound II eluting faster in the hydrophilic aqueous phase

**Fig. 3:**
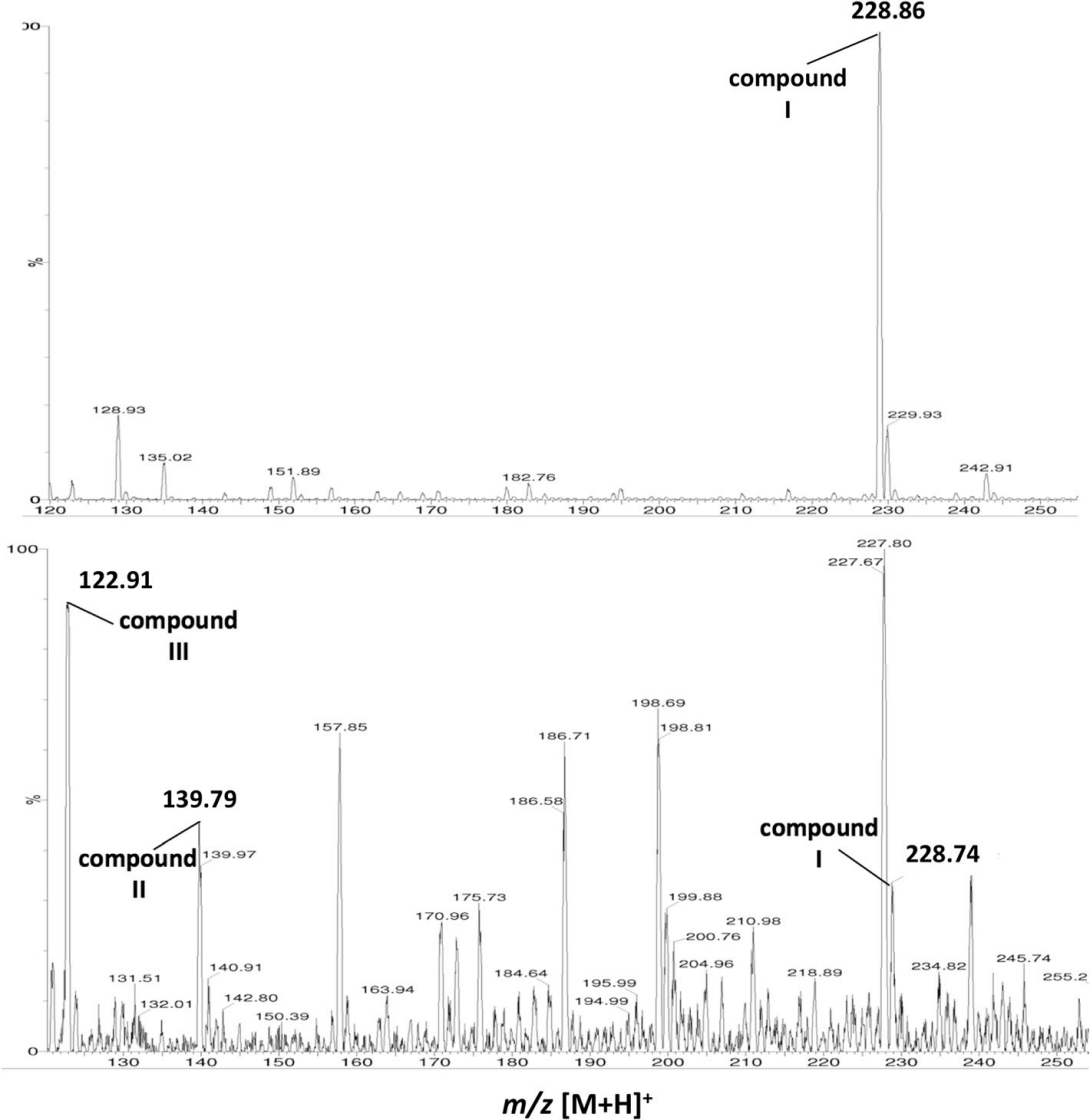
Mass spectroscopic analysis of PbLSD treated resveratrol following thin-layer chromatography analysis. Samples of resveratrol were either treated with *Pb*LSD, or not, and then analyzed by thin-layer chromatography. Areas of the thin-layer chromatography matrix including the substrate and products were scraped together from each lane and profiled by mass spectroscopy. Top panel: sample from untreated resveratrol control lane. Bottom panel: sample from PbLSD treated resveratrol lane

Enzymatic activity of the purified GST-tagged *Pb*LSD was determined to evaluate the preference for stilbene and carotenoid substrates (Fig. 2a). The well-characterized *At*CCD1 enzyme from *Arabidopsis* was included as a control. The incubation of resveratrol (compound I) with *Pb*LSD yielded two product bands by thin-layer chromatography analysis, coincident with the complete disappearance of the substrate band (Fig. 2b, left panel), while *At*CCD1 yielded no products from resveratrol. In contrast, incubation of lutein (compound IV; Fig. 2b, right panel) with *At*CCD1 yielded a product band just above the lutein substrate band, with similar migration and staining properties to that observed previously for the expected 4,9-dimethyldodeca-2,4,6,8,10-pentaene-1,12-dial (compound VII; a C14 dialdehyde) product following cleavage at the 9′-10′ and 9–10 double bonds [22]. However, no significant product band was generated by incubation of lutein with *Pb*LSD suggesting no detectable activity against this C40 carotenoid in vitro. No activity was detected for *Pb*LSD against any other C40 carotenoids tested in vitro either (data not shown). Further HPLC analyses of the reaction of *Pb*LSD with resveratrol yielded two profile product peaks (Fig. 2c) with retention times consistent with the benzaldehyde products detected for the in vitro reaction of *N. romaticivorans* NOV1 and NOV2 enzymes with resveratrol using the same protocol [36]. Mass spectrometric analysis (Fig. 3) of the resveratrol-derived products scraped from thin-layer chromatography plates, confirmed the expected molecular weights for the production of 3,5-dihydroxybenzaldehyde (compound II; *m/z* [M + H]^+^ 139) and 4-hydroxybenaldehyde (compound III; *m/z* [M + H]^+^ 123). It is notable that compound (II) is apparently present at lower concentrations in all assays, possibly due to its higher volatility.

### In vivo enzymatic activities of recombinant PbLSD

**Fig. 4:**
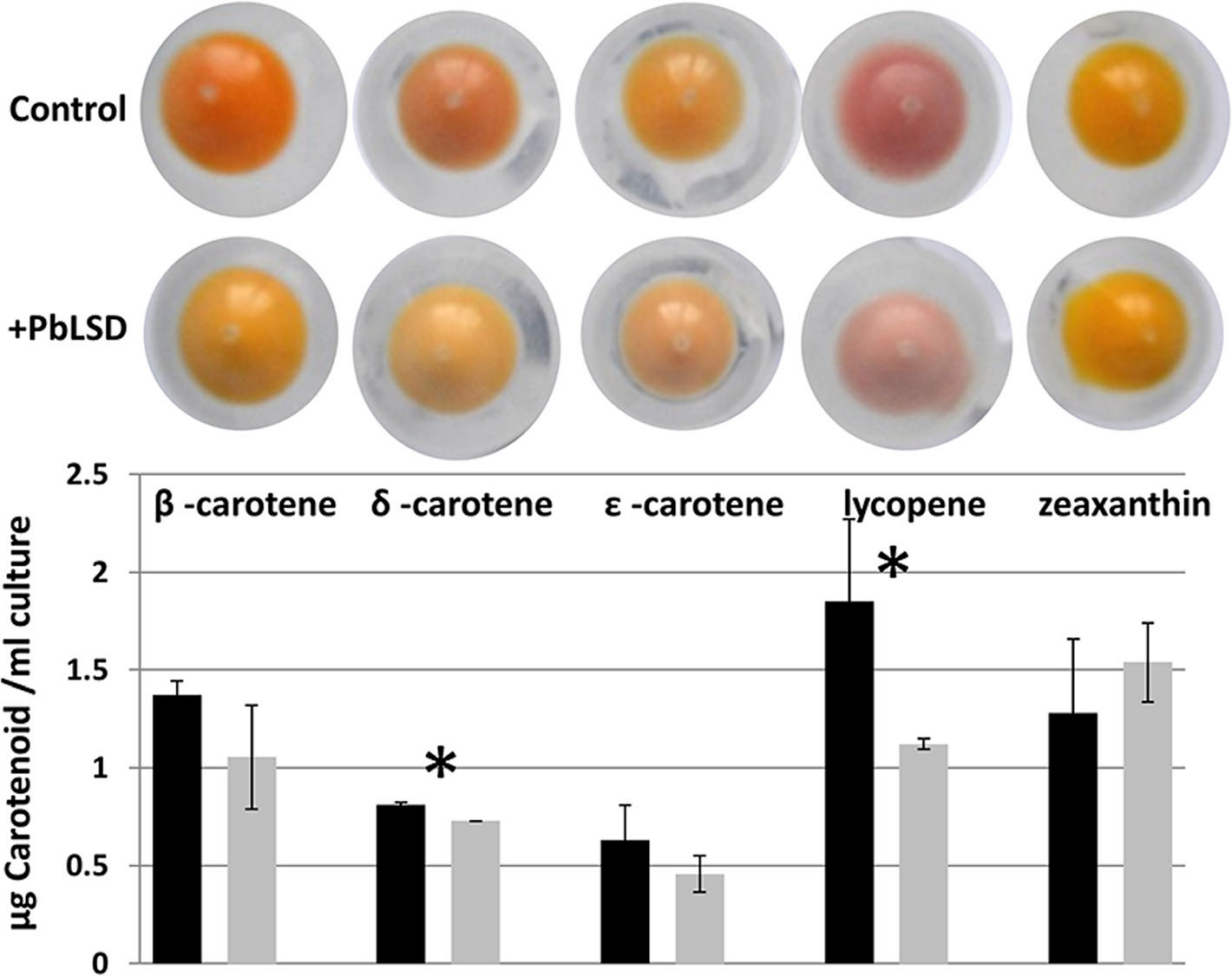
Carotenoid cleavage by *Pb*LSD in recombinant carotenoid producing *E.coli* strains. Top panel: The effect of expression of *Pb*LSD in select carotenoid accumulating *E. coli* strains transformed with pET28b + -*Pb*LSD and induced with IPTG was assessed visually (bottom row of pellets). β-, δ- or ε- carotene are produced by co-expression from pAC-BETA, pAC-DELTA and pAC-EPSILON, respectively, lycopene by co-expression from pAC-LYC and zeaxanthin by co-expression from pAC-ZEAX. Negative controls include strains transformed with only the pAC vectors, but induced with IPTG (Control; top row of pellets). Bottom panel: Quantitative analysis of carotenoid accumulation in liquid-grown cultures. Samples labeled *Pb*LSD and Control are extracts from representatives of the samples shown in the top panel. Relative carotenoid concentration values are shown as an average of three samples each. Significant differences (*p* values < 0.05) were assessed by student T test and are denoted with an *

Because CCOs have been reported to lose as much as 75% of their activity during purification [36–39], the lack of any detectable *Pb*LSD activity against lutein and other carotenoids in vitro does not preclude the possibility of it having lower levels of activity against carotenoids in vivo. To check this possibility, in vivo assays of *Pb*LSD activity, in which the enzyme was expressed in carotenoid-accumulating strains of *E. coli* where enzymatic activity can be assessed by changes in color and quantified by substrate depletion spectrophotometrically, were carried out. Visual inspection revealed that induction of expression of *Pb*LSD caused some decreases in the coloration of δ-carotene and lycopene containing cell pellets (Fig. 4, top panel). These effects were confirmed by spectrophotometric quantitation, which showed statistically significant decreases in the accumulation of these two carotenoids. The effect was most notable for the straight chain lycopene, which showed a > 40% decrease in carotenoid accumulation. While β- and ε- carotenes also showed decreases in their accumulation in the *Pb*LSD cell pellets visually, the changes were much smaller both visually and spectrophotometrically, and these were not found to be significant by student T-test analysis. Finally, consistent with the lack of any detectible visual change in the coloration of the zeaxanthin containing pellets, quantification also showed no decrease in zeaxanthin levels in the presence of *Pb*LSD. Attempts at isolating products from these in vivo assays have not, to date, yielded sufficient product for identification. This is consistent with the very low levels of activity observed herein and in earlier studies that have attributed low product accumulation in vivo to volatility and further catabolism of the products [22, 40]. Overall these results suggest the possibility that *Pb*LSD may have at least some weak carotenoid cleaving oxygenase activity against selected carotenoids, in addition to its much stronger stilbene cleaving oxygenase activity. However, validation of this carotenoid cleaving activity awaits product determination, and any potential biological relevance of this weak activity also remains enigmatic.

### Crystal structure of recombinant *Pb*LSD

To gain additional insight into molecular factors mediating these functional observations, a structural investigation was initiated. The purified *Pb*LSD crystallized in the monoclinic space group P2_1_ and a molecular replacement solution containing four monomers in the asymmetric unit was obtained using a subunit of *Synechocystis* apocarotenoid-15,15′-oxygenase (ACO; 30% sequence identity, PDB: 2BIW). Sections of the chains, particularly those containing looped regions with greatest deviation from the sequence of ACO, required considerable manual rebuilding, but the final structure was refined to 1.9 Å with R and R_free_ values of 16.3 and 19.4% respectively (Table 1). In two monomers, the ordered protein contained six and two residues, respectively, of the His-tag expression sequence at the N-terminus, while, in the other two, the ordered structure began at Ser2. All monomers presented a continuous main chain to the C-terminal Gly481 and could be superimposed with an average root mean squared deviation of the main chain atoms in the six combinations of 0.26 Å. Size exclusion chromatography of the affinity enriched enzyme yielded no evidence of dimeric or higher order oligomers (data not shown), suggesting the affinity-purified enzyme is likely in a monomeric form for crystallization, as well as in in vitro assays.

**Fig. 5:**
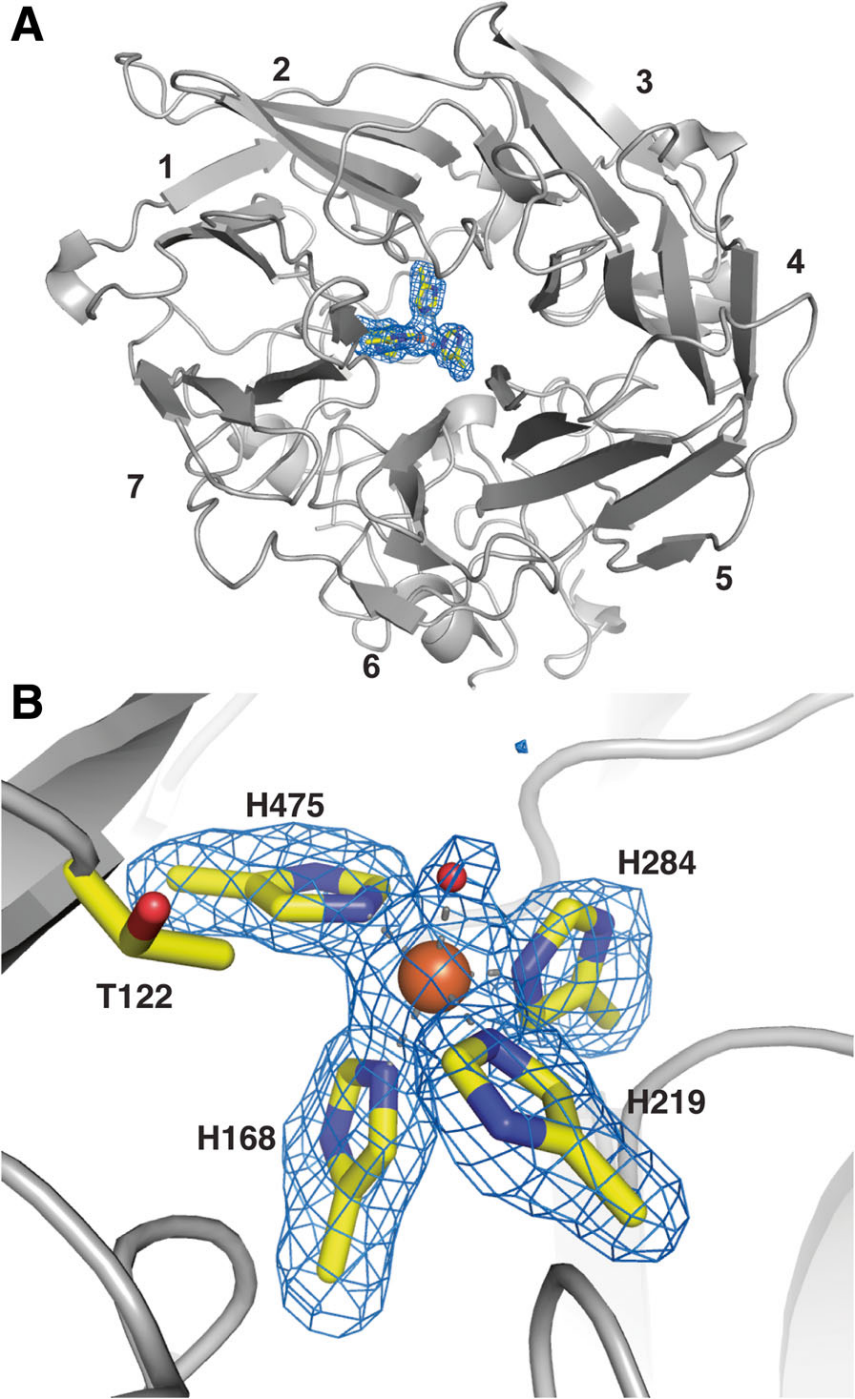
View of the *Pb*LSD seven-blade propeller fold (**a**) 4-His-Fe unit (**b**)**.** In panel **a**, the single subunit is oriented to show the seven blades (numbered) with the electron density of the 4-His-Fe unit visible in the center. In panel **b**, the subunit is rotated to view the 4-His-Fe unit modeled into the F_o_-F_c_ omit electron density map. The F_o_-F_c_ omit electron density maps in panels **a** and **b** at 7.0 σ were calculated without the four histidines, iron and the waters in the model. The water forming the 5th coordination site, is represented by a small ball surrounded by density in the image, while the iron is represented by a larger central ball. Thr122 is proposed to occlude the 6th Fe coordination site

The secondary structure of *Pb*LSD is composed primarily of β strands, 27 in total, organized in a seven-bladed β-propeller motif with blades comprised of 3, 4 or 5 β strands (Fig. 5a). Despite the addition of EDTA to the protein purification protocol, density representing what is most likely a single iron atom at the reaction center of each monomer, is observed and secured by four histidine residues at a distance of 2.1 Å and has a fifth ligand association to a water or hydroxide also at 2.1 Å (Fig. 5b). This active site organization is consistent with that observed across the broader family of SCOs, CCOs and NCEDs for which crystal structures are available. That the density at the catalytic center most probably represents iron, is supported by the activity detected against resveratrol in vitro (Figs. 2 and 3), without any need for metal-chelation and reconstitution with iron. The sixth metal-ligand coordinating position, is occluded by a nearby (4.4 Å) threonine (T122) methyl group. A similar unit in lipoxygenases from plant (PDB: 3PZW) and bacteria (PDB: 4G32) has the Fe-water complex sequestered at all five ligand sites by three His residues, one Asn residue and the C-terminal carboxylate, while in the human version (PDB: 4NRE) the Asn is replaced by a second hydroxide or water. There is, however, no other structural similarity between oxygenases and lipoxygenases, with the latter containing primarily α-helical secondary structure.

The recently published structure of SCO NOV1 was shown convincingly to contain dioxygen associated with the iron atom [13], which provided a clear insight into the reaction mechanism. The electron density adjacent to the iron atoms in the maps of *Pb*LSD was reviewed with this in mind, including the calculation of omit maps lacking water at the fifth-ligand position and with dioxygen in the model. In three of the four subunits, the density clearly resembled and was satisfied by water, but in the fourth, the density resembled dioxygen in shape but was satisfied by a single oxygen atom, suggesting partial occupancy. The fifth ligand is therefore reported as water in all four subunits.

**Fig. 6:**
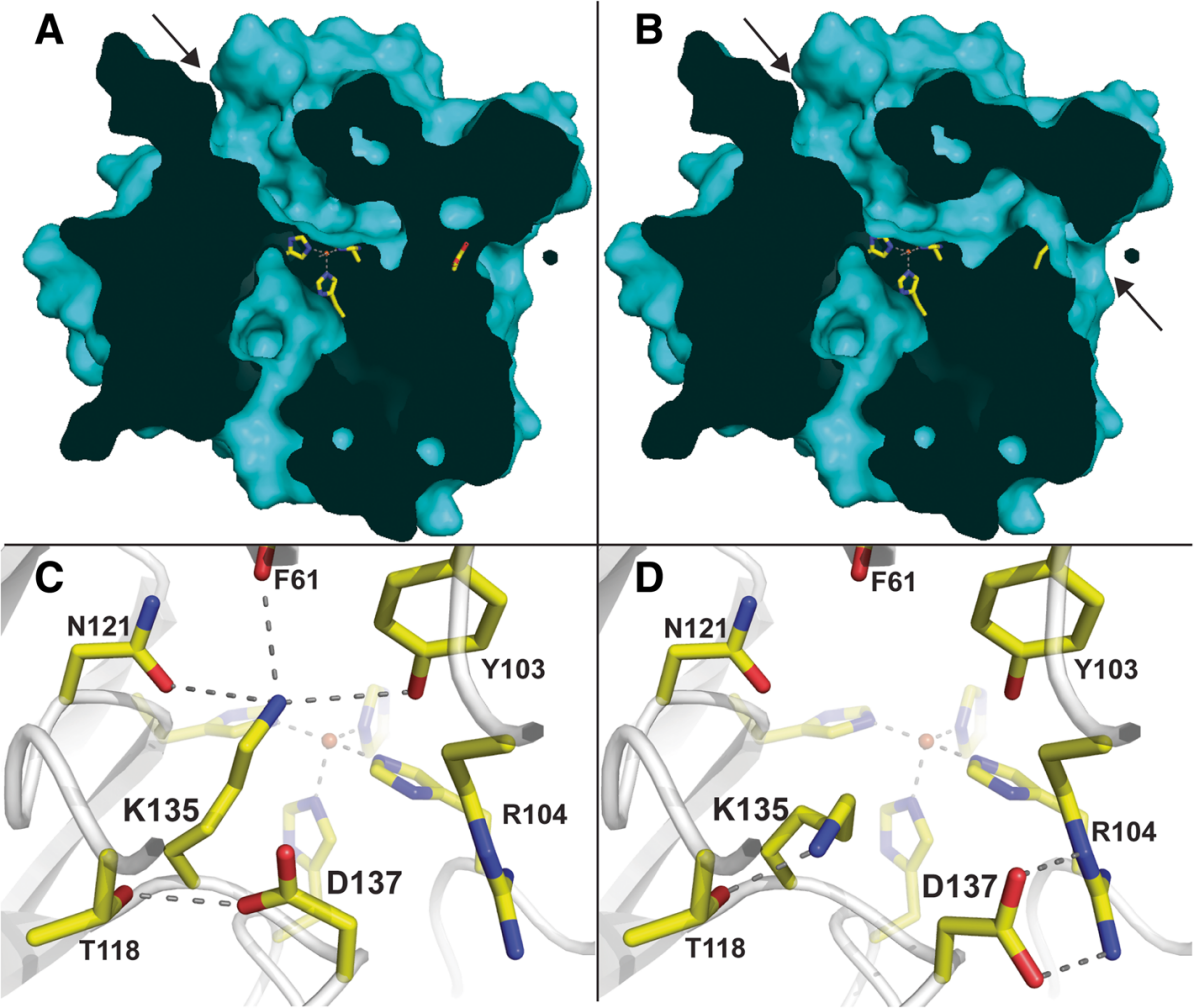
Active site cavities in *Pb*LSD. **a** A surface slice of the derived crystal structure. The arrow is highlighting the classic stilbene substrate access cavity in the native *Pb*LSD structure, from which product is expected to exit. **b** A surface slice of the derived crystal structure highlighting the tunnel cavity that can also exist within *Pb*LSD by rotation of the side chains of amino acids Lys135 and Arg137, consistent with tunnel cavities in other carotenoid cleaving enzymes. Due to these images being produce by ‘slicing’, the side chain of Lys135 is not visible (its on the side of the slice that was cut off), but the side chain of Asp137 is visible blocking the channel in panel **a** and rotated away in panel **b**. Representations of the region surrounding Lys135 and Arg 137, highlighting the different molecular interactions observed are shown in (**c**) the native structure and (d) the native structure with Lys 135 and Arg 137 rotated to form a tunnel through the enzyme

The structures of ACO and *Pb*LSD can be superimposed with an rmsd of 1.6 Å for 405 of the 488 main chain residues. The 83 other residues yielded higher rmsd deviations, likely due to occurring primarily in random coil regions. Similarly, that of NOV1 can be superimposed with *Pb*LSD with a rmsd of 1.4 Å over a similar range, highlighting the structural similarity within the broader family of CCO enzymes. One striking structural feature of these proteins generally is the existence of cavities and even tunnels extending deep into the protein [10, 13]. Consistent in all CCO structures, including now *Pb*LSD (Fig. 6a), is a cavity extending from under the 4-His-Fe center, down and out to the surface of the enzyme. A possible role as an access route for molecular oxygen required during catalysis has been proposed for this cavity [10]. The relevance of the remaining cavities and/or tunnels generally lies with substrate access and product release, and thus specificity and selectivity of the enzymes [13]. In the case of *Pb*LSD, the cavity on the left in Fig. 6a (highlighted by an arrow), and extending from the surface diagonally down to the catalytic site, is similar to that observed in the SCO NOV1 structure [13]. It is proposed to serve as both the access route for the stilbenes into the binding pocket and the exit route for any products that are released from the active site. The existence of this cavity in *Pb*LSD is consistent with the in vitro activity it displayed against resveratrol (Figs. 2 and 3).

How this substrate cavity, as depicted in Fig. 6a, might account for the weak activity of *Pb*LSD against longer carotenoids observed in vivo (Fig. 4) is not obvious. However, it is possible to extend the existing substrate cavity into a continuous tunnel spanning the width of the enzyme (Fig. 6b) by a simple rotation of the side chains of just two amino acids, Lys135 and Asp137. The orientations and interactions of the two residues are compared in Fig. 6c and d, respectively before and after rotation. The interactions formed after rotation suggest relatively stable conformers, particularly with two interactions at 2.4 and 3.1 Å between Asp137 and Arg104 (Fig. 6d) compared with a single 2.8 Å hydrogen bond between Asp137 and Thr118 before rotation (Fig. 6c). In addition, Lys135 rotates from making three long, at 3.3, 3.4 and 3.2 Å, interactions respectively with Asn121, Tyr103 and the carbonyl of Phe61 (Fig. 6c), to making a single 2.4 Å hydrogen bond with Thr118 (Fig. 6d). Despite the apparent favorability of rotation, no structural variability in this region was noted among the subunits in the crystal structure. However, this tunnel would be consistent with the architecture observed in the ACO enzyme that evolved to accommodate larger substrates [10, 13]. In ACO, a large hydrophobic cavity (equivalent of right side of enzymes in Fig. 6a and b) extends as a narrow tunnel from the surface to the active site central cavity. Interactions with carotenoid substrates are enabled here by a proposed membrane interaction region on the surface proximal to this large hydrophobic cavity. Any products are proposed to exit through a second tunnel out the other side of the enzyme (equivalent to left side in Fig. 6a and b). Ultimately, whether the observed weak in vivo carotenoid cleaving activity of *Pb*LSD might be enabled by induction/stabilization of such a tunnel conformation through interactions with higher concentrations of substrates, cellular membranes and/or homo-dimerization of the enzyme remains to be determined.

### In silico substrate docking

Computational docking to the derived crystal structure was performed to investigate possible protein-ligand interactions involved in substrate binding and the determination of substrate preferences*.* Maestro was used to dock resveratrol and an array of carotenoid targets using the XP Glide program [34]. The primary metric used by Maestro for comparison of the binding affinities of docked substrates is the “GlideScore”. This metric empirically scores generated models, approximating ligand binding free energy (with more negative values indicating tighter binding) and is based on parameters that are important in substrate binding, including but not limited to optimization of electrostatics and hydrophobic enclosure of ligands. Sorting the best pose of each substrate docked within *Pb*LSD yielded preferred binding partners with lutein, 9-*cis*-violaxanthin, and neoxanthin as the three substrates with the highest theoretical/predicted affinities (Table 2). It is interesting to note that although there was near-complete in vitro cleavage of resveratrol (Figs. 2 and 3), binding to *Pb*LSD was calculated as weaker than the docked carotenoids. This may be a reflection of its smaller size and also the need for some elasticity during catalysis.

**Table 2:** XP Glide calculated binding affinities for an array of molecules virtually docked to *Pb*LSD

Substrate	GlideScore
Lutein	−8.376
9-*cis*-violaxanthin	−8.308
Neoxanthin	−7.539
Zeaxanthin	−6.964
β-citraurol	−6.825
β-carotene	−5.442
Lycopene	−4.638
Resveratrol	−4.479

**Fig. 7:**
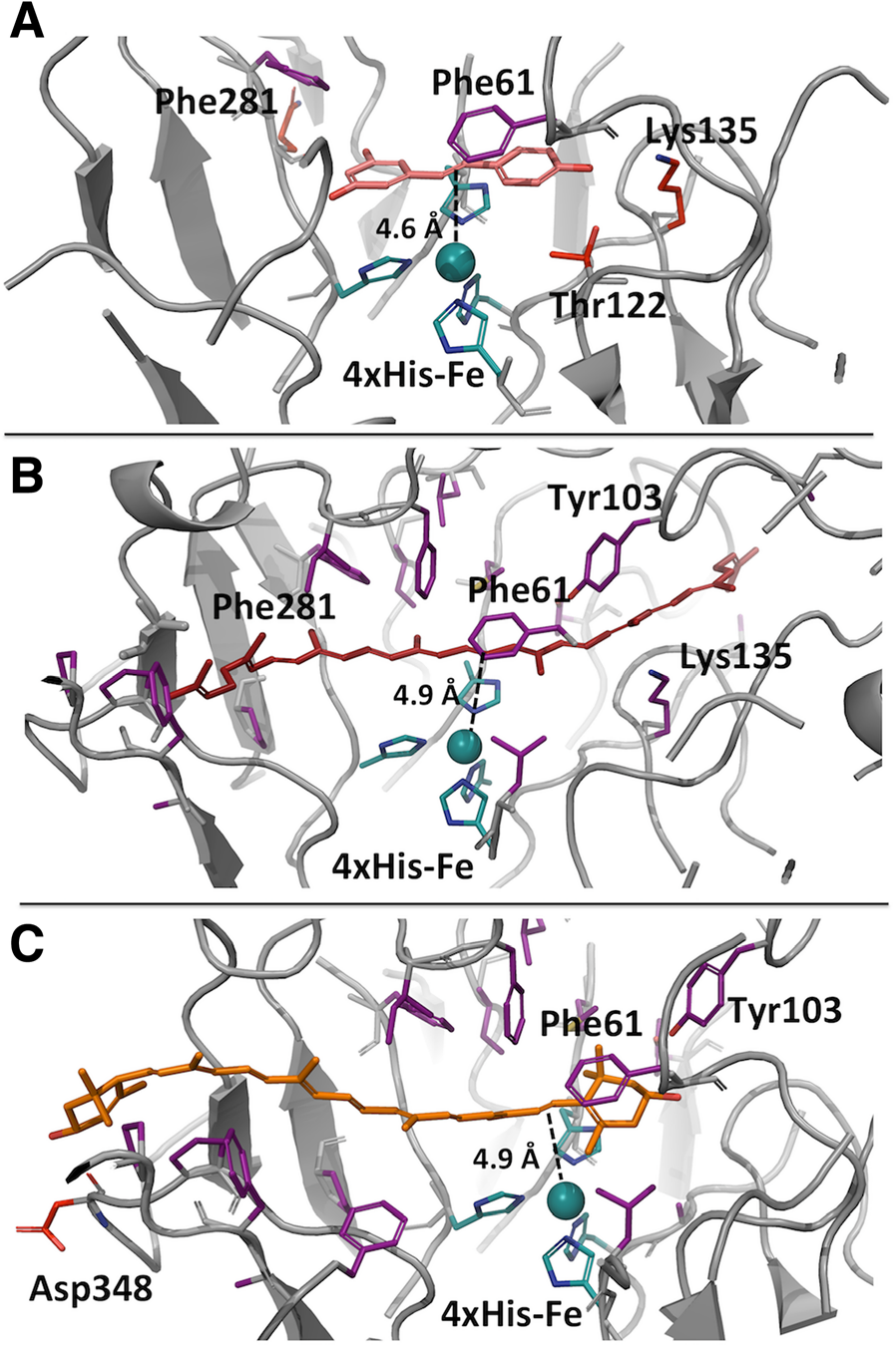
Virtual docking of molecules in the binding pocket of the crystal structure of enzyme *Pb*LSD. A cartoon representation of the active site of *Pb*LSD docked in proximity to the catalytic site 4-His-Fe subunit. **a** Resveratrol (**b**) lycopene and (**c**) lutein, all with the double bond most proximal to the catalytic center indicated by a dotted line (15–15′ and 7′-8′ respectively for lycopene and lutein). Potential pi-stacking and hydrogen bonding partners are labelled

The predicted locations of substrate binding of the highest scored models were then examined for protein-ligand interactions and for orientation relative to the active center. Resveratrol was docked in the cavity with its single cleavable double bond (Fig. 2a) directly above the Fe center, at a distance of 4.6 Å (Fig. 7a), consistent with the enzyme’s in vitro activity against the substrate (Fig. 2). Key interactions include hydrogen bonding between residues Thr122, Lys135, and the backbone oxygen of Gln282 to the hydroxyl groups of resveratrol. Further, π-stacking interactions within the model were provided by residues Phe61 and Phe281. These interactions, as well as the co-ordination of the iron center, are all consistent with those observed in the co-crystal structure of NOV1 with resveratrol (PDB: 5J54) [13].

While not as optimal as the resveratrol interaction, at 4.9 Å from the Fe-center, the 15–15′ double bond of lycopene is highlighted as a likely target for oxidative cleavage by *Pb*LSD (Fig. 7b). Indeed, in silico, the lycopene chain is able to extend in through the stilbene substrate cavity, over the catalytic center and continue into a fairly narrow cleft adjacent to Lys135. A small shift of < 0.5 Å in the main chain atoms from Leu134 to Leu139 and small rotations in the side chains of Lys135 and Asp137 were all that were required to accommodate the lycopene molecule. This is consistent with the proposed opening of the tunnel achieved by small rotations of the same two residues (Fig. 6b). The tightness of the channel raises the question of whether retention of carotenoid cleavage products within the enzyme might be an alternate possible explanation for the lack of product detected in our functional assays.

Interestingly, despite having the most favorable GlideScore, and thus strongest theoretical binding affinity for *Pb*LSD, lutein did not extend fully into the cavity, and neither its central double bond, nor the 9′-10′ double bond approached the Fe-center (Fig. 7c). This general orientation was consistent across all bi-cyclic C40 carotenoids modelled (data not shown). Presumably the large, modified cyclohexene ring at either end prevents further entrance into the cavity due to steric hindrance/and or hydrogen bonding with Tyr103. That the 7′-8′ double bond of lutein is situated in closer proximity to the active center (4.9 Å; much as for lycopene’s 15–15′ double bond), might suggest a possible target for the bicyclic carotenoid cleavage activity detected in vivo. However validation of the cleavage of lutein awaits product characterization, which as discussed above and elsewhere remains an ongoing challenge [22, 40].

## Conclusions

The activity of a lignostilbene-α,β-dioxygenase orthologue from *P. brassicacearum* has been characterized, showing in vitro activity against resveratrol as a primary substrate. Interestingly, weak putative cleavage activity against a number of carotenoids, most notably lycopene, was also detected in in vivo recombinant carotenoid expressing *E. coli* experiments. The structure of the enzyme determined by X-ray crystallography, and refined to 1.9 Å, contains the conserved seven-bladed β-propeller and 4-His-Fe(II) motifs characteristic of carotenoid and stilbene cleavage oxygenases. As well, the expected SCO-like substrate cavity was clearly evident, and its extension into a longer tunnel extending through the protein to potentially accommodate longer carotenoids was achieved by modelling small rotations in just two side chains. Further in silico modelling highlighted an obvious lignostilbene binding site, as well as possible carotenoid binding sites that could enable their cleavage in the absence of a tunnel. While the carotenoid cleaving activity of this enzyme remains to be validated by product identification, the potential differential activities reported here for PbLSC in vivo compared to in vitro are reflected in other CCOs [36–39], and together emphasize the need to understand the relationship between these enzymes and their environment, as it pertains to enzyme oligomerization, membrane interactions, substrate accessibility and specificity.

## Abbreviations

CCD: carotenoid cleavage dioxygenase
CCO: carotenoid cleavage oxygenases
IPTG: isopropyl-ß-D-thiogalactopyranoside
LSD: lignostilbene-α,β-dioxygenase
NCED: 9-*cis*-epoxycarotenoid dioxygenase
*Pb*: *Pseudomonas brassicacearum*
SCO: stilbene cleaving oxygenase
